# Supplementary material for: HIF1α-regulated glycolysis promotes activation-induced cell death and IFN-γ induction in hypoxic T cells
Source: Nat Commun. 2024 Oct 30;15:9394. doi: 10.1038/s41467-024-53593-8 (PMC11526104; doi:10.1038/s41467-024-53593-8)
Supplement: Supplementary file 1 — Supplementary Information [file 41467_2024_53593_MOESM1_ESM.pdf]

Supplementary Information

HIF1α-glycolysis engages activation-induced cell death to drive IFN-γ induction in hypoxic T cells

Hongxing Shen[1], Oluwagbemiga A. Ojo[1], Haitao Ding[1], Logan J. Mullen[2], Chuan Xing[1], M. Iqbal Hossain[3], Abdelrahman Yassin[1], Vivian Y. Shi[1], Zach Lewis[1], Ewa Podgorska[1], Shaida A. Andrabi[3], Maciek R. Antoniewicz[4], James A. Bonner[1,5], and Lewis Zhichang Shi[1,3,5,6,*]

[1]Department of Radiation Oncology, Heersink School of Medicine, University of Alabama at Birmingham (UAB-SOM), Birmingham, AL 35233, USA.

[2]Genomics Core Laboratory, Institute of Arctic Biology, University of Alaska Fairbanks, Fairbanks, Alaska, 99775, USA.

[3]Department of Pharmacology and Toxicology, UAB-SOM, USA
[4]Department of Chemical Engineering, University of Michigan, Ann Arbor, MI 48109, USA.
[5]O'Neal Comprehensive Cancer Center, UAB-SOM, USA
[6]Department of Microbiology and Immunology Institute, UAB-SOM, USA

*Correspondence author: Lewisshi@uabmc.edu.

Supplemental Figures 1-8

Figure S1. HIF1α but not HIF2α controls IFN-γ induction in hypoxic T cells, *in vitro*.
Figure S2. HIF1α-glycolysis drives IFN-γ induction in hypoxic T cells.
Figure S3. Direct regulation of IFN-γ induction in hypoxic T cells by HIF1α and intracellular acetyl CoA, *in vitro*.
Figure S4. Reduced IFN-γ production in *Hif1α*$^{-/-}$ T cells is not due to proliferative defect.
Figure S5. Attenuated AICD in *Hif1α*$^{-/-}$ T cells is not mediated by extrinsic factors.
Figure S6. HIF1α controls effector function of TILs.
Figure S7. HIF1α in T cells governs therapeutic effects of ICB.
Figure S8 FACS gating strategies.

Supplemental Tables 1-3

Supplemental Table 1. List of flow antibodies used in this manuscript.
Supplemental Table 2. List of Western Blot antibodies used in this manuscript.
Supplemental Table 3. List of real-time PCR primers used in this manuscript.

Supplemental Fig. 1

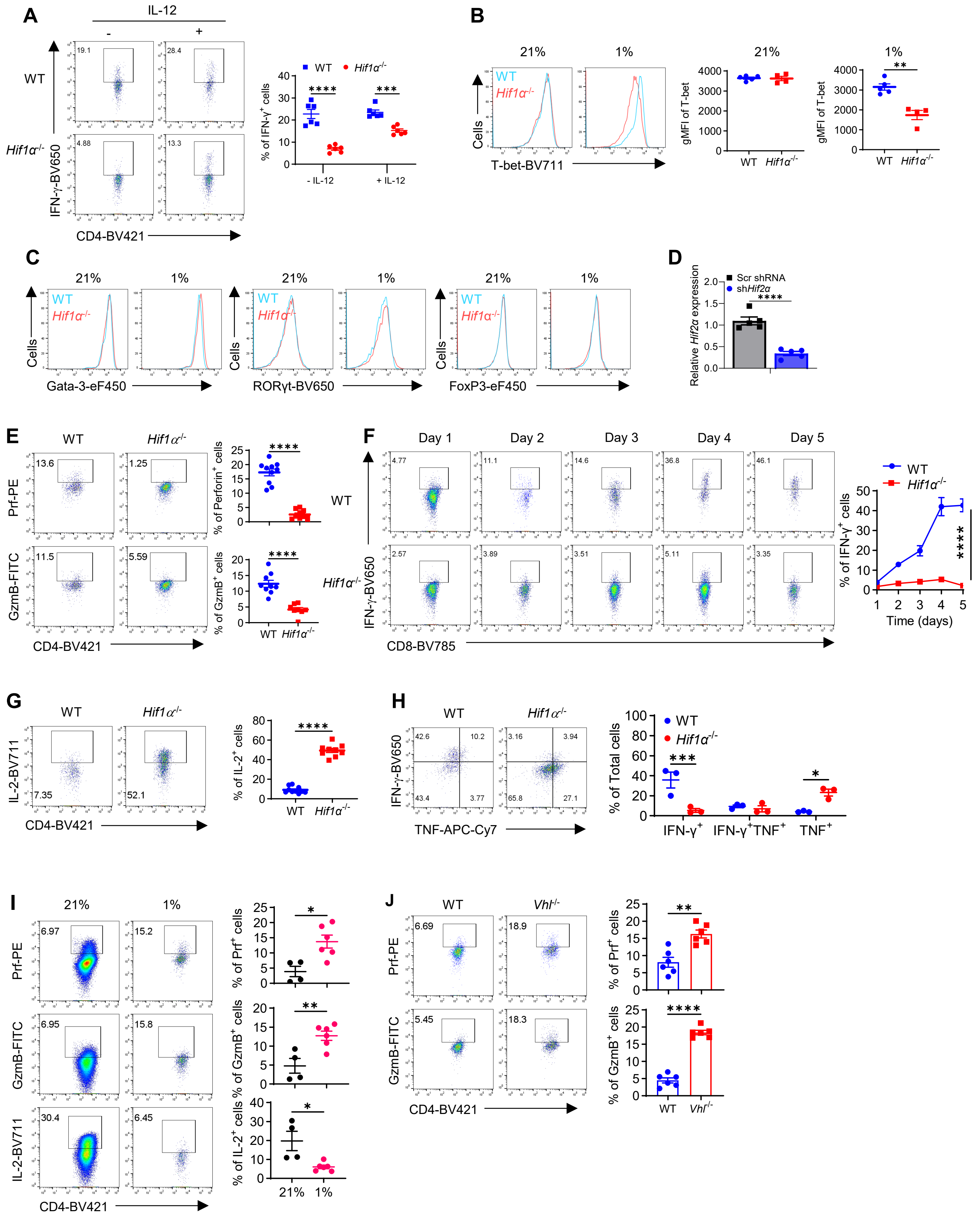

Figure S1. *Hif1α* but not *Hif2α* controls IFN-γ induction in hypoxic T cells, *in vitro.*

A. Naïve CD4⁺ T cells isolated from WT and *Hif1α*$^{-/-}$ mice were activated under hypoxia for 5 days, with or without IL-12, followed by IFN-γ detection ($N = 6$, ****$p < 0.0001$, ***$p = 0.0003$). **B-C**. Histogram overlays of T-bet (**B**) and Gata-3, RORγt and FoxP3 (**C**) expression in WT ($N = 5$) and *Hif1α*$^{-/-}$ ($N = 4$) $CD4^+$ T cells, activated under normoxia and hypoxia for 2 days (**$p = 0.0013$). **D**. Activated $CD4^+$ T cells were transduced with retroviruses expressing scrambled shRNA (Scr shRNA) or *shHif2α*, followed by RT-PCR to check *Hif2α* mRNA expression ($N = 5$, ****$p < 0.0001$). **E**. Production of perforin (Prf) and granzyme B (GzmB) in WT and *Hif1α*$^{-/-}$ $CD4^+$ T cells activated in hypoxia for 5 days ($N = 10$, ****$p < 0.0001$, ***$p = 0.0003$, *$p = 0.013$). **F**. Naïve WT ($N = 3$) and *Hif1α*$^{-/-}$ ($N = 6$) $CD8^+$ T cells were activated under hypoxia (1% O_2). IFN-γ production was assessed daily from Day 1-5 (****$p < 0.0001$). **G-H**. IL-2 (**G**) ($N = 10$) and IFN-γ^+TNF$^+$ (**H**) ($N = 3$) by WT and *Hif1α*$^{-/-}$ $CD4^+$ T cells activated in hypoxia for 5 days (****$p < 0.0001$, ***$p = 0.0003$, *$p = 0.013$). **I**. Production of Prf (*$p = 0.0112$), GzmB (**$p = 0.0063$), and IL-2 (*$p = 0.0112$) in $CD4^+$ T cells activated under normoxia (21% O_2, $N = 4$) and hypoxia (1% O_2 , $N = 6$) for 5 days. **J**. Production of Prf (**$p = 0.0012$) and GzmB (****$p < 0.0001$) in WT ($N = 6$) or *Vhl*$^{-/-}$ ($N = 6$) $CD4^+$ T cells activated under hypoxia for 5 days. A two-sided Student's t-test was used in **B, D, F-G, I-J** for statistical analyses. Two-way ANOVA with Šídák's multiple comparisons test (with adjustment) was used for **A**, **E** and **H**. All the experiments were repeated at least twice. Pooled results shown in the dot plots and bar graphs depicted means ± SEM for all samples in each group, with each dot denoting an independent sample. Source data are provided as a Source Data file.

Supplemental Figure 2

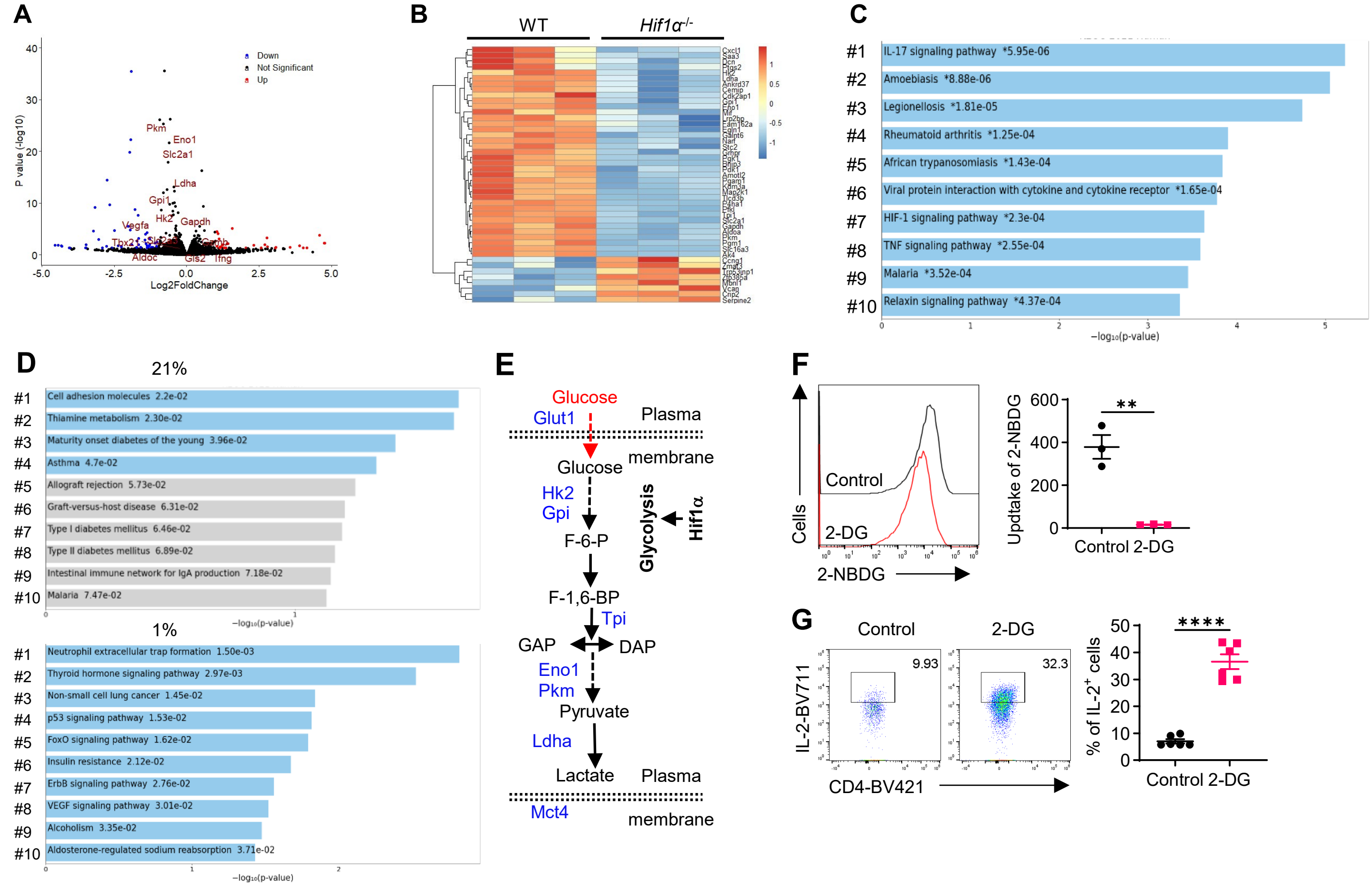

Fig. S2 ***Hif1α*-glycolysis drives IFN-γ induction in hypoxic T cells.**
A-C. Total RNAs extracted from WT and *Hif1α*$^{-/-}$ CD4^{+} T cells activated under normoxia for 48h were subjected to RNA-Seq. The gene expression analyses were performed using DESeq2 (version 1.34.0). The Wald test was used to calculate the *p* values and log2 fold changes. Genes with an adjusted *p* value < 0.05 and absolute log2 fold change > 1 were considered as differentially expressed genes (DEGs). A volcano plot was used to show all upregulated and downregulated DEGs using the ggplot2 R package (**A**), with top 50 identified DEGs shown in a heatmap (**B**). Top 10 enriched signaling pathways (downregulated) from Enriched Kyoto Encyclopedia of Genes and Genomes (KEGG) analyses of DEGs were shown in (**C**). Significant terms of the KEGG pathways were selected with a p value <0.05. **D**. Significantly upregulated top 10 enriched signaling pathways in *Hif1α*$^{-/-}$ CD4^{+} T cells activated under normoxia (21% O_2) (Upper Panel) and hypoxia (1% O_2) (Lower Panel). **E.** A diagram of the glycolytic pathway, with the major glycolytic genes highlighted in blue. **F**. Uptake of 2-NBDG by activated naïve CD4 T cells treated with solvent (Control) or 0.5 μM of 2-DG (*N* = 3 per group, ***p* = 0.0027). **G**. IL-2 production by WT CD4^{+} T cells activated under hypoxia for 5 days, treated with solvent (Control) or 0.5 μM of 2-DG (*N* = 6 per group, *****p* < 0.0001). A two-sided Student's t-test was used in **F** and **G** for statistical analyses. All the experiments were repeated at least twice. Pooled results shown in the dot plots and bar graphs depicted means ± SEM for all samples in each group, with each dot denoting an independent sample. Source data are provided as a Source Data file.

Supplemental Figure 3.

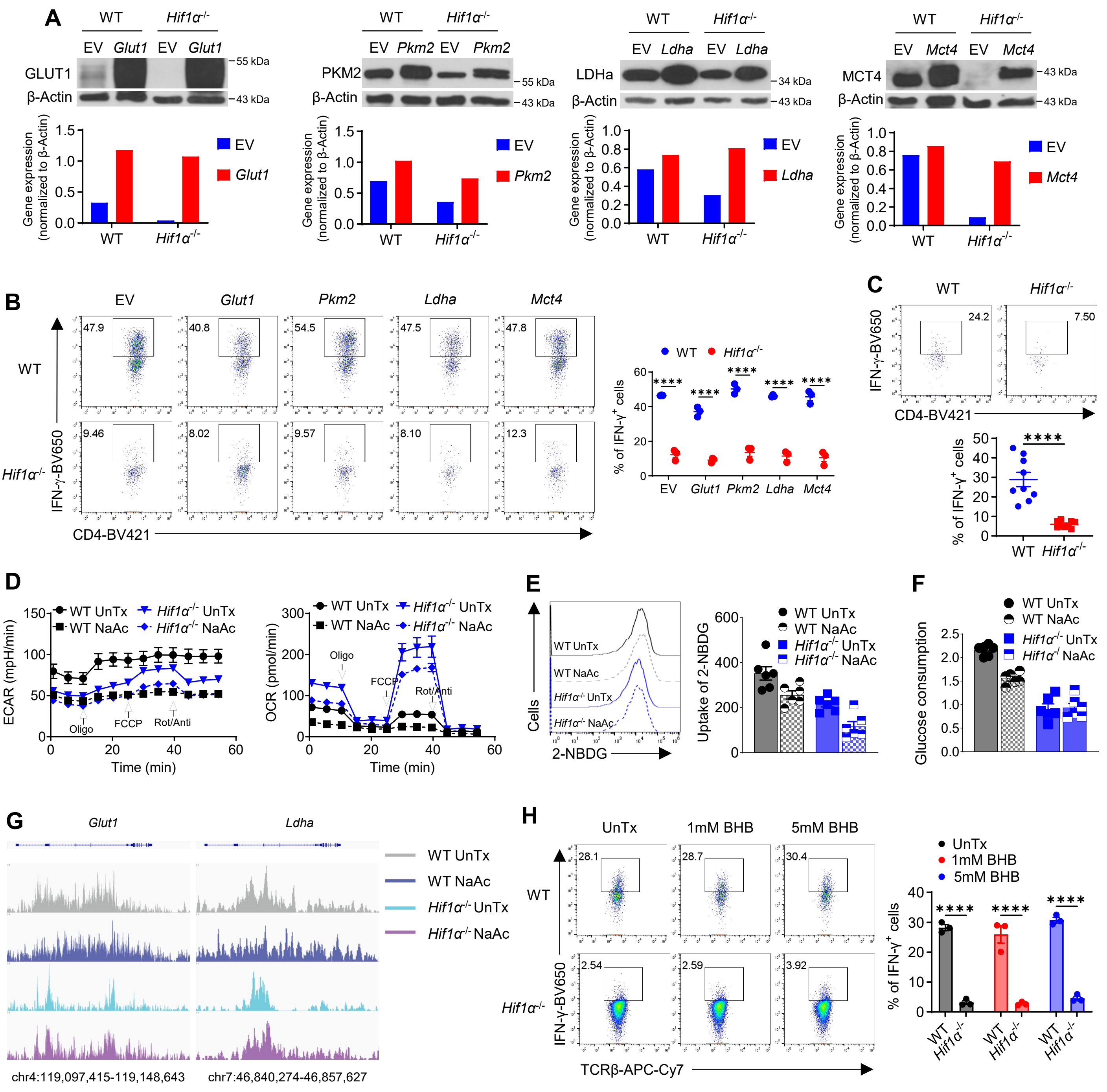

Figure S3. Direct regulation of IFN-γ induction in hypoxic T cells by *Hif1α* and intracellular acetyl CoA, *in vitro.*

A. Protein expression of GLUT1, PKM2, LDHa and MCT4 in activated WT and *Hif1α*$^{-/-}$ CD4^{+} T cells successfully transduced (GFP^{+}) with empty retroviruses (EV) or retroviruses expressing *Glut1*, *Pkm2*, *Ldha, or Mct4*. **B.** GFP^{+} T cells from (**A**) were activated under hypoxia for 5 days and analyzed for IFN-γ production *(N* = 3 per group, ****$p < 0.0001$). **C**. IFN-γ production by activated WT and *Hif1α*$^{-/-}$ CD4^{+} T cells co-cultured with MB49 cells for 48h, under hypoxia (*N* = 9 per group, ****$p < 0.0001$ by two-sided Student's t-test). **D-E**. Extracellular acidification rate (ECAR) and oxygen consumption rate (OCR) (**D**), uptake of 2-NBDG (**E**), and glucose consumption (**F**) *(N* = 6 per group) by activated WT and *Hif1α*$^{-/-}$ CD4^{+} T cells treated with solvent (UnTx) or 20 mM of NaAc. **G.** H3K9Ac enrichment at *Glut1* and *Ldha* loci in activated WT and *Hif1α*$^{-/-}$ CD4^{+} T cells treated with solvent (UnTx) or 20 mM of NaAc. **H**. Activated WT and *Hif1α*$^{-/-}$ CD4^{+} T cells under hypoxia were treated with solvent (UnTx), 1mM, or 5mM BHB for 48h and analyzed for IFN-γ production *(N* = 3 per group, ****$p < 0.0001$). Two-way ANOVA with Šídák's multiple comparisons test (with adjustment) was used for **B** and **G**. All the experiments were repeated at least twice. Pooled results shown in the dot plots and bar graphs depicted means ± SEM for all the samples in each group. Source data are provided as a Source Data file.

Supplemental Figure 4

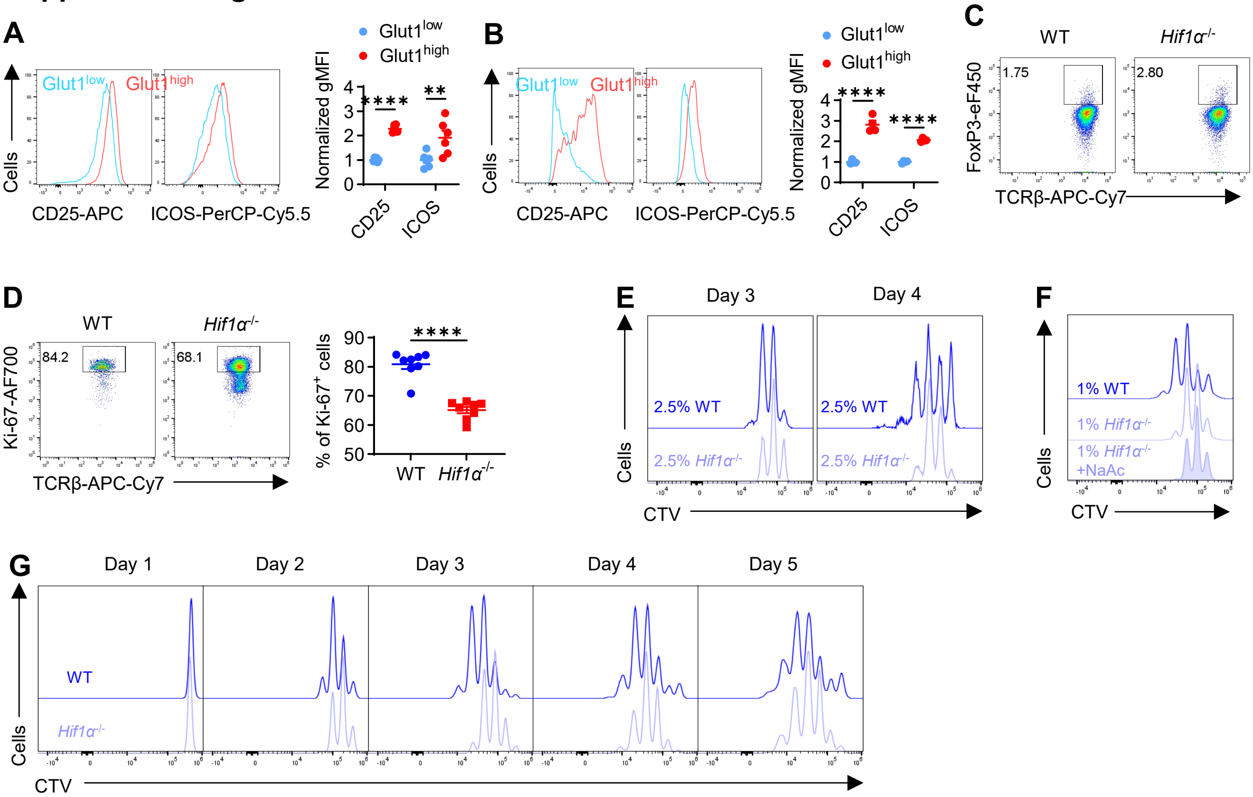

Figure S4. Reduced IFN-γ production by *Hif1α*$^{-/-}$ T cells is not due to proliferative defect.
A-B. Naïve WT CD4^{+} (**A**) (*N* = 6 per group, ****p <0.0001, **p = 0.0011) and CD8^{+} (**B**) (*N* = 4 per group, ****p <0.0001) T cells were activated under hypoxia for 3 days. Gated live Glut1^{low} and Glut1^{high} T cells were analyzed for the expression of ICOS and CD25 (gMFI: geometric mean fluorescence intensity). **C-D**. Naïve WT and *Hif1α*$^{-/-}$ CD4^{+} T cells activated under hypoxia were analyzed for FoxP3 (**C**) (*N* = 4 per group) and Ki-67 (**D**) (*N* = 8 per group, ****p <0.0001 by two-sided Student's t-test). **E**. CellTrace Violet (CTV)-labeled naïve WT and *Hif1α*$^{-/-}$ CD4^{+} T cells were activated in 2.5% O_2. CTV dilution were detected on day 3 and day 4 following activation. **F**. CTV dilution in activated WT and *Hif1α*$^{-/-}$ CD4^{+} T treated without or with NaAc (Day 3). **G**. CTV-labeled naïve WT and *Hif1α*$^{-/-}$ CD8^{+} T cells were activated under hypoxia. CTV dilution were detected daily from Day 1-5. Two-way ANOVA with Šídák's multiple comparisons test (with adjustment) was used for **A** and **B**. All the experiments were repeated at least twice. Pooled results shown in the dot plots depicted means ± SEM for all the samples in each group, with each dot denoting an independent sample. Source data are provided as a Source Data file.

Supplemental Figure 5

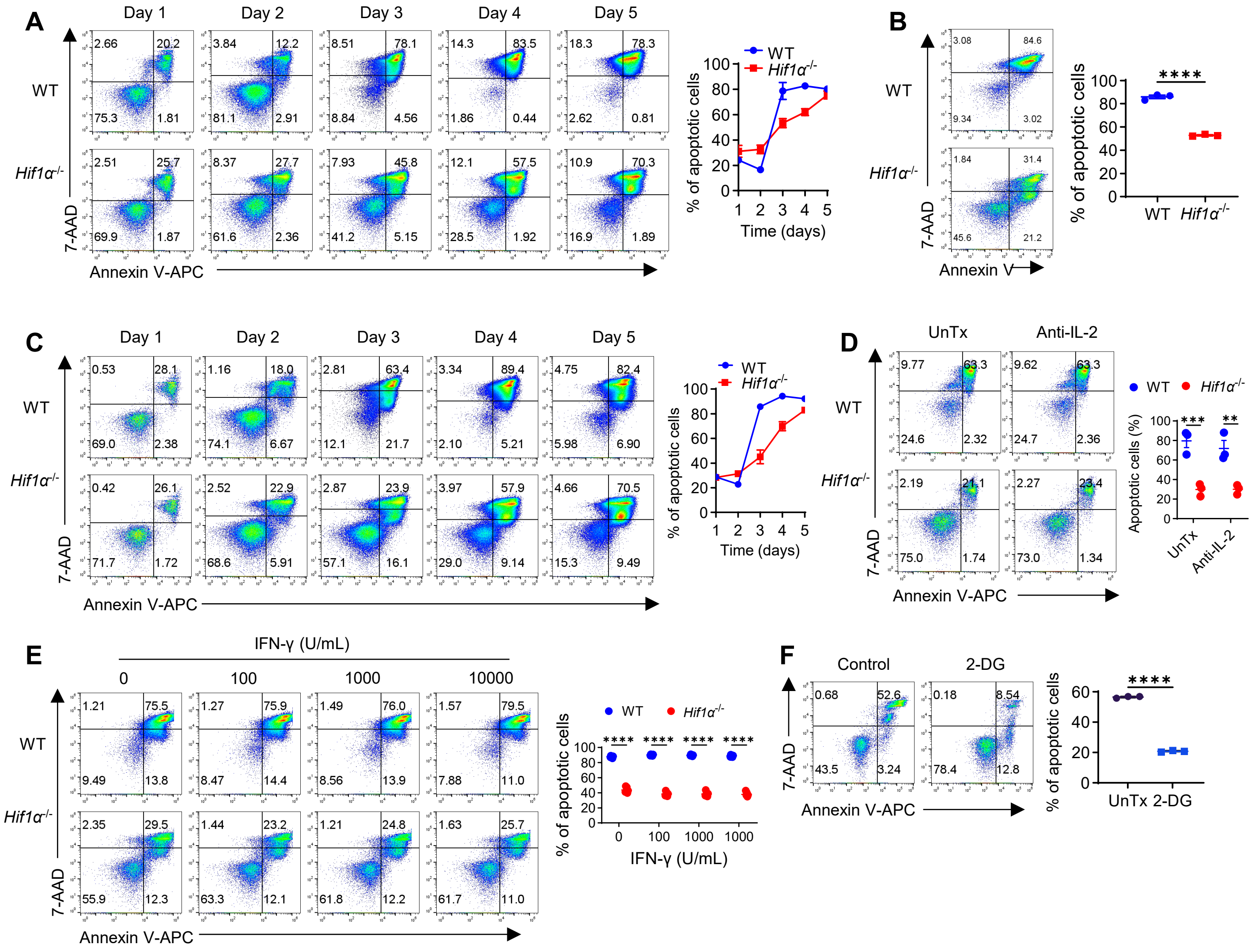

Figure S5. Attenuated AICD in *Hif1α*$^{-/-}$ T cells is not mediated by extrinsic factors.
A. Naïve WT CD4^{+} T cells were activated under hypoxia (1% O_2) and assessed cell death daily from Day 1-5 by staining for 7-AAD and Annexin V. The line graph on the right showed percentages of apoptotic cells. **B**. Naïve WT CD4^{+} T cells were activated under 2.5% O_2 for 3 days to assess cell death (*****p* < 0.0001). **C.** Assays in (**A)** were repeated but with 50% of old media replaced by fresh media every day from Day 1-4. **D-E**. Naïve WT and *Hif1α*$^{-/-}$ CD4^{+} were activated under hypoxia for 3 days, with or without blocking antibodies against IL-2 (**D)** (****p* = 0.0006, ***p* = 0.002), and with or without added IFN-γ (**E**) (*****p* < 0.0001) followed by analyses of cell death by 7-AAD and Annexin V staining. **F**. Naive human CD4^{+} T cells isolated from PBMCs of healthy donors were activated under hypoxia for 3 days, in the absence and presence of 0.5mM 2-DG, followed by 7-AAD and Annexin V staining to detect cell death (*****p* < 0.0001). *N* = 6 per group in **A** and **C**; *N* = 3 per group in **B, D, E** and **F**. A two-sided Student's t-test was used in **B** and **F** for statistical analyses. Two-way ANOVA with Šídák's multiple comparisons test (with adjustment) was used for **D** and **E.** All the experiments were repeated at least twice. Pooled results shown in the dot plots depicted means ± SEM for all the samples in each group, with each dot denoting an independent sample. Source data are provided as a Source Data file.

Supplemental Figure 6

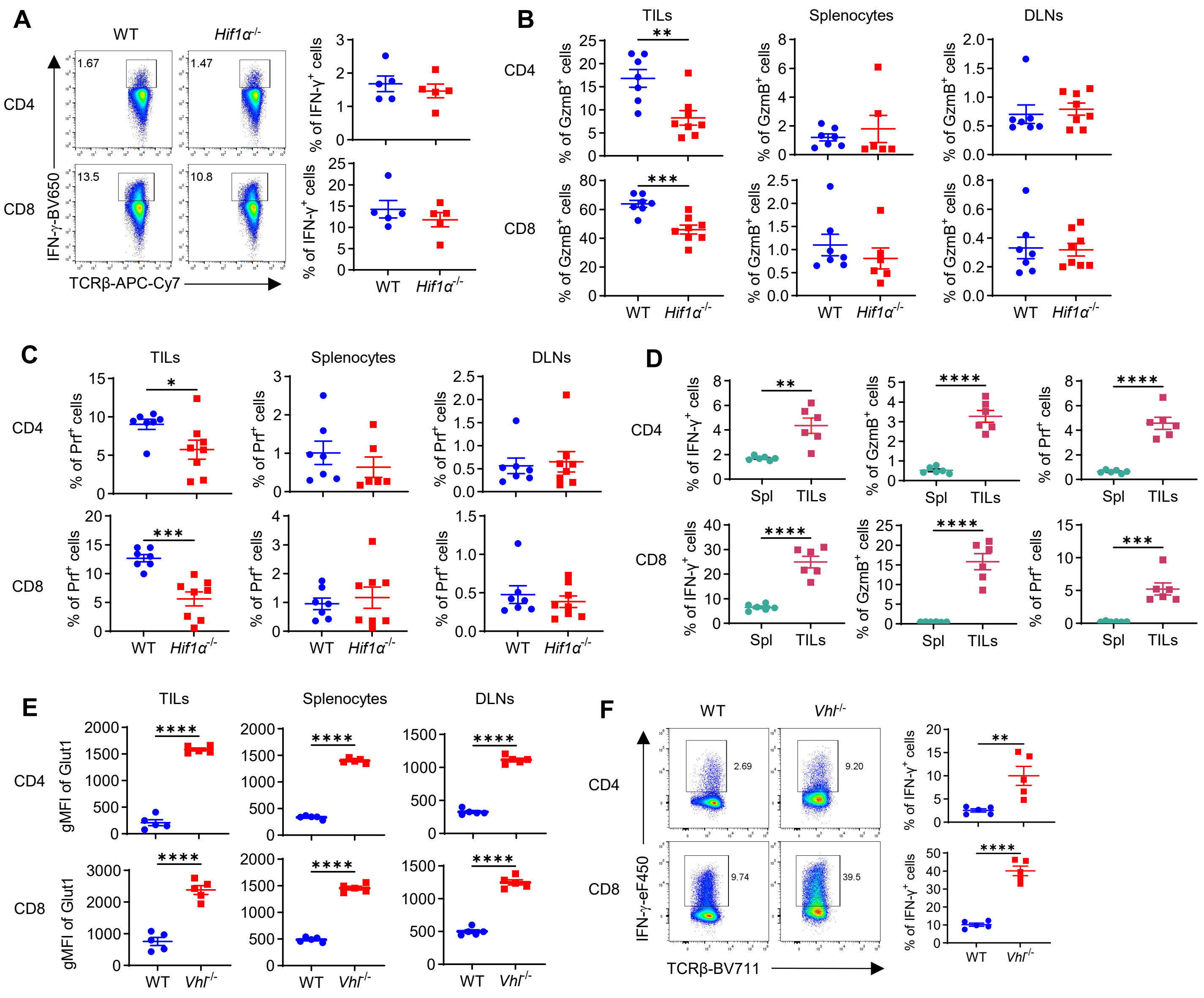

Figure S6. *Hif1α* controls effector function of TILs.
A. T cells isolated from draining lymph nodes (DLNs) of WT or *Hif1α*$^{-/-}$ mice bearing established MB49 bladder tumor were analyzed for IFN-γ production (*N* = 5 per group). **B-C**. Production of GzmB (**B**) (*N* = 7 per group, $^{**}p = 0.0041$, $^{***}p = 0.0007$) and Prf (**C**) (*N* = 7 per group, $^{*}p = 0.0425$, $^{***}p = 0.0003$) by CD4$^+$ and CD8$^+$ TILs or CD4$^+$ and CD8$^+$ T cells from spleen and DLNs of WT or *Hif1α*$^{-/-}$ mice bearing established MB49 bladder tumor. **D**. Production of IFN-γ, GzmB, and Prf by CD4$^+$ and CD8$^+$ splenocytes versus CD4$^+$ and CD8$^+$ TILs, isolated from WT mice bearing MB49 bladder tumor (*N* = 6 per group, $^{**}p = 0.0016$, $^{***}p = 0.0003$, $^{****}p < 0.0001$). **E-F**. T cells isolated from WT or *Vhl*$^{-/-}$ mice bearing established MB49 bladder tumor were analyzed for Glut1 expression (**E**) (*N* = 5 per group, $^{****}p < 0.0001$) in CD4$^+$ and CD8$^+$ TILs, splenocytes, and DLNs, as well as IFN-γ production by DLN CD4$^+$ and CD8$^+$ T cells (**F**) (*N* = 5 per group, $^{**}p = 0.007$ $^{****}p < 0.0001$). A two-sided Student's t-test was used in **A-F** for statistical analyses. All the experiments were repeated 2-5 times. Pooled results shown in the dot plots depicted means ± SEM for all the mice in each group, with each dot denoting an individual mouse. Source data are provided as a Source Data file.

Supplemental Figure 7

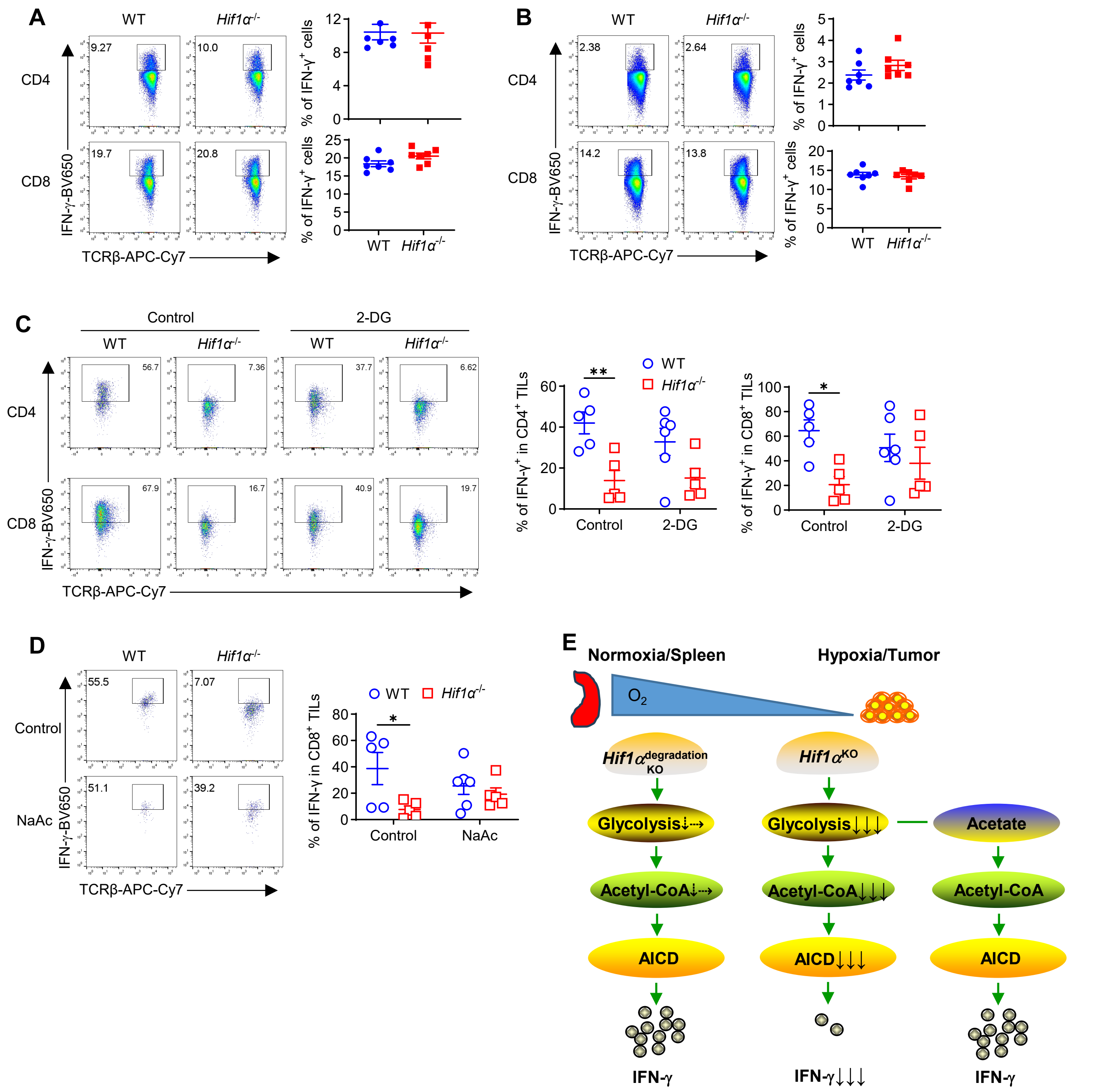

Figure S7. *Hif1α* in T cells governs therapeutic effects of ICB.
A-B. WT and *Hif1α*$^{-/-}$ mice bearing palpable MB49 bladder tumor were treated with combined anti-CTLA-4+anti-PD-1 (ICB). IFN-γ production by CD4$^+$ and CD8$^+$ T cells from spleens (**A**) (N = 7 per group) and draining lymph nodes (DLNs) (**B**) (N = 7 per group) was shown. **C**. IFN-γ production by CD4$^+$ and CD8$^+$ TILs from MB49 bladder tumor-bearing WT and *Hif1α*$^{-/-}$ mice treated with ICB alone or in conjunction with administration of 2-DG (N = 5 per group, $^{**}p$ = 0.006, $^*p$ = 0.0183). **D**. IFN-γ production by CD8$^+$ TILs from MB49 bladder tumor-bearing WT and *Hif1α*$^{-/-}$ mice treated with ICB alone or in conjunction with administration of sodium acetate (NaAc) (N = 5 per group, $^*p$ = 0.0277). **E**. A proposed model of how T cell-intrinsic *Hif1α*-glycolysis controls IFN-γ production in hypoxic T cells. Two-way ANOVA with Šídák's multiple comparisons test (with adjustment) was used for **C-D**. All the experiments were repeated 2-5 times. Pooled results shown in the dot plots depicted means ± SEM for all the mice in each group, with each dot denoting an individual mouse. Source data are provided as a Source Data file.

Supplementary Figure 8 FACS gating strategies

FACS strategy 1: for splenocytes, DLN, and cultured cells.

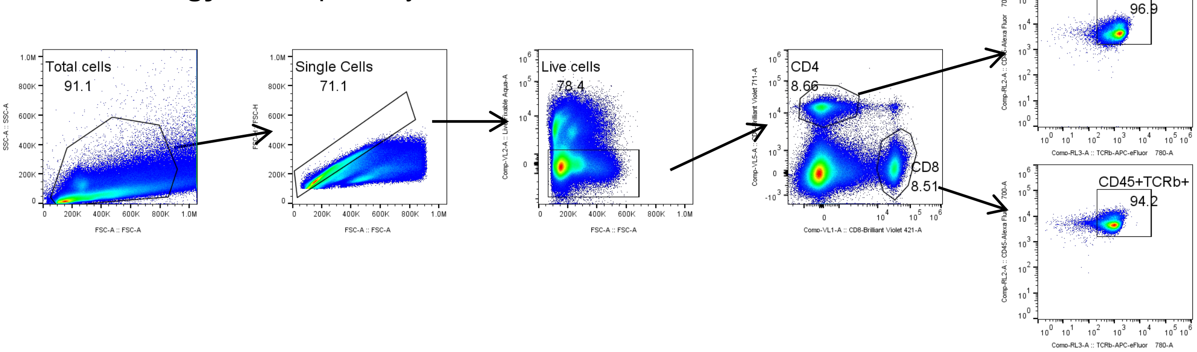

FACS strategy 2. for single cell suspensions of tumors.

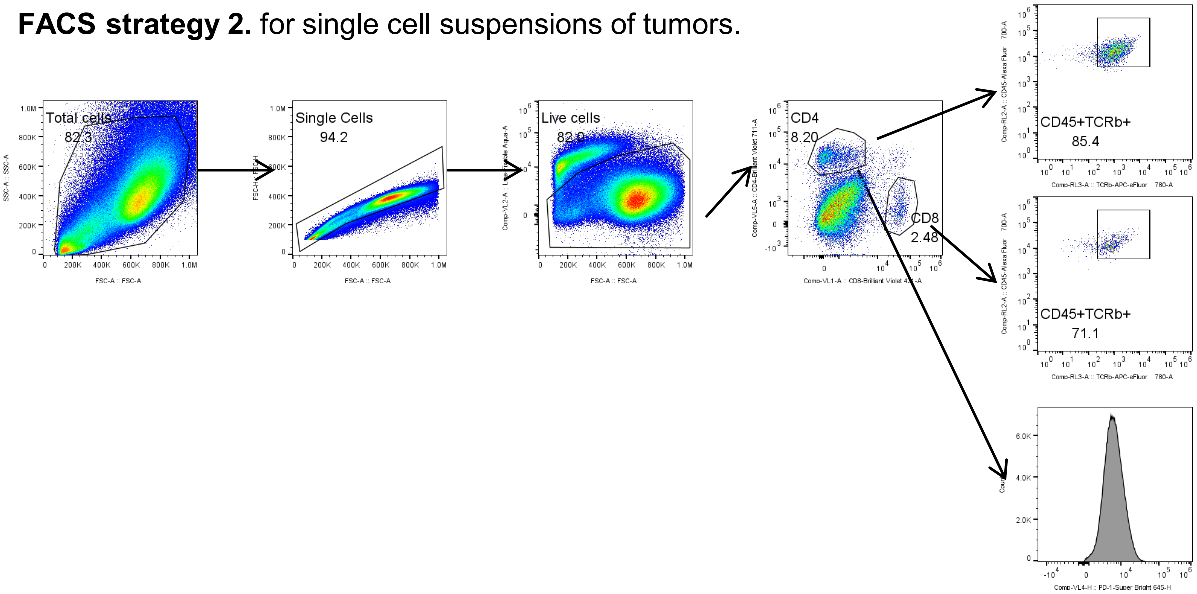

Supplementary Figure 8 FACS gating strategies

FACS strategy 1 was used in all panels from Figures 1-5, 6A, 6C, 6E. Supplemental Figures S1-S5, S6A, S6F, S7A and S7B .

FACS strategy 2 was used in Figures 6B, 6D, 6F, 7C, 7G, Supplemental Figures S7C and S7B.

Supplemental Table 1. List of flow antibodies used in this manuscript.

Antibodies	Clone	Source	Identifier	Antibody dilution
Anti-Mouse CD8 Brilliant Violet 785™	53-6.7	BD Biosciences	#563332	1/200
Anti-Mouse CD4 Brilliant Violet 421™	RM4-5	BioLegend	#100544	1/200
Anti-Mouse CD45 PerCP-Cyanine5.5	30-F11	Thermo Fisher	#45-0451-82	1/200
Anti-Mouse CD45.1 PerCP-Cyanine5.5	A20	BioLegend	#110728	1/200
Anti-Mouse CD45.2 Alexa Fluor® 700	104	BioLegend	#109822	1/200
Anti-Mouse TCRβ APC-Cy7	H57-597	BioLegend	#109220	1/200
Anti-Mouse Perforin PE	S16009A	BioLegend	#154306	1/100
Anti-Mouse Granzyme B FITC	QA16A02	BioLegend	#372206	1/100
Anti-Mouse IFN-γ Brilliant Violet 650™	XMG1.2	BioLegend	#505832	1/100
Anti-Mouse IL-2 Brilliant Violet 711™	JES6-5H4	BioLegend	#503837	1/100
Anti-Mouse T-bet Brilliant Violet 711™	4B10	BioLegend	#644820	1/100
Anti-Mouse ICOS PerCP-Cyanine5.5	7E.17G9	BioLegend	#117424	1/200
Anti-Mouse CD25 APC	PC61.5	Thermo Fisher	#17-0251-82	1/200
Anti-Mouse FoxP3 eFluor™ 450	FJK-16s	Thermo Fisher	#48-5773-82	1/100
Anti-Mouse HIF1α APC	Mgc3	Thermo Fisher	#17-7528-82	1/100
Anti-Mouse Ki-67 Alexa Fluor® 700	SolA15	Thermo Fisher	#56-5698-82	1/100
Anti-Mouse RORγt Brilliant Violet 650™	Q31-378	BD Biosciences	# 564722	1/100
Anti-Human/Mouse Gata-3 eFluor™ 660	TWAJ	Thermo Fisher	# 50-9966-42	1/100
Anti-Human/Mouse Glut1 Alexa Fluor® 405	EPR3915	Abcam	#ab210438	1/100
Anti-Human CD3 Super Bright™ 702	OKT3	Thermo Fisher	#67-0037-42	1/200
Anti-Human IFN-γ Brilliant Violet 605™	B27	BD Biosciences	#562974	1/100
Anti-Human CD4 Brilliant Violet 650™	L200	BD Biosciences	#563737	1/200

Supplemental Table 2. List of Western Blot antibodies used in this manuscript.

Name	Clone	Source	Catalog	Antibody dilution
HIF-1α	D2U3T	Cell Signaling Technology	14179	1/1000
HIF-2α	E2N9W	Cell Signaling Technology	57921	1/1000
Glut1	polyclonal	Sigma	07-1401	1/1000
Hk2	C64G5	Cell Signaling Technology	2867	1/1000
Ldha	Polyclonal	Cell Signaling Technology	2012	1/1000
Pkm2	D78A4	Cell Signaling Technology	4053	1/1000
Mct4	D-1	Santa Cruz Biotechnology	sc-376140	1/1000
PDH	C54G1	Cell Signaling Technology	3205	1/1000
phospho-PDH (Ser293)	E4V9L	Cell Signaling Technology	37115	1/1000
β-Actin	C-4	Santa Cruz Biotechnology	sc-47778 HRP	1/10000

Supplemental Table 3. List of real-time PCR primers used in this manuscript.

Gene name	Forward primer	Reverse primer
Hif1α	AGCTTCTGTTATGAGGCTCACC	TGACTTGATGTTCATCGTCCTC
Hif2α	TGAGTTGGCTCATGAGTTGC	TTGCTGATGTTTTCCGACAG
Glut1	CAGTTCGGCTATAACACTGGTG	GCCCCCGACAGAGAAGATG
Hk2	TGATCGCCTGCTTATTCACGG	AACCGCCTAGAAATCTCCAGA
Gpi	TCAAGCTGCGCGAACTTTTTG	GGTTCTTGGAGTAGTCCACCAG
Tpi1	CCAGGAAGTTCTTCGTTGGGG	CAAAGTCGATGTAAGCGGTGG
Pkm2	GCCGCCTGGACATTGACTC	CCATGAGAGAAATTCAGCCGAG
Eno1	TGCGTCCACTGGCATCTAC	CAGAGCAGGCGCAATAGTTTTA
Ldha	CATTGTCAAGTACAGTCCACACT	TTCCAATTACTCGGTTTTTGGGA
Mct4	TCACGGGTTTCTCCTACGC	GCCAAAGCGGTTCACACAC
Actin	CATTGCTGACAGGATGCAGAAGG	TGCTGGAAGGTGGACAGTGAGG
