# Supplementary material for: HIF1α-regulated glycolysis promotes activation-induced cell death and IFN-γ induction in hypoxic T cells
Source: Nat Commun. 2024 Oct 30;15:9394. doi: 10.1038/s41467-024-53593-8 (PMC11526104; doi:10.1038/s41467-024-53593-8)
Supplement: Supplementary file 2 — Description of Additional Supplementary Files [file 41467_2024_53593_MOESM2_ESM.pdf]

Description of Additional Supplementary Files

Supplementary Data 1. RNA-Seq-Hypoxia WT versus Hif1a-KO

Total RNAs extracted from WT and Hif1$\alpha^{-/-}$ $CD4^+$ naïve T cells activated under hypoxia for 48h were subjected to RNA-Seq. The gene expression analyses were performed using DESeq2 (version 1.34.0). The two-sided Wald test was used to calculate the p values and log2 fold changes. Genes with an adjusted p value < 0.05 and absolute log2 fold change > 1 were considered as differentially expressed genes (DEGs). A volcano plot was used to show all upregulated and downregulated DEGs using the ggplot2 R package.

Supplementary Data 2. RNA-Seq-Normoxia WT versus Hif1a-KO

Total RNAs extracted from WT and Hif1$\alpha^{-/-}$ $CD4^+$ naïve T cells activated under normoxia for 48h were subjected to RNA-Seq. The gene expression analyses were performed using DESeq2 (version 1.34.0). The two-sided Wald test was used to calculate the p values and log2 fold changes. Genes with an adjusted p value < 0.05 and absolute log2 fold change > 1 were considered as differentially expressed genes (DEGs). A volcano plot was used to show all upregulated and downregulated DEGs using the ggplot2 R package.

Supplementary Data 3. ^{13}C metabolic flux analysis results based on fitting of data from tracer experiments with 74% [U-^{13}C] glucose.

Depicted were the estimated net and exchange fluxes (nmol/10^6 cells/hr) in WT and Hif1$\alpha^{-/-}$ naïve $CD4^+$ T cells activated overnight in regular culture medium containing 5 mM glucose and then supplemented with 15 mM of [U-^{13}C]glucose for additional 6 and 24h. Samples (supernatants and cell lysates) were collected at the designated time points and analyzed as described in the Methods. Accurate 95% confidence intervals of fluxes were determined by evaluating the sensitivity of the minimized SSR to flux variations (Antoniewicz et al., 2006).

Supplementary Data 4. Cut&Run data WT versus Hif1a-KO

H3K9Ac enrichment in activated WT and Hif1$\alpha^{-/-}$ T cells treated with solvent (UnTx) or 20 mM of NaAc. Two-sided glmQLFTest was used for statistical analysis.
