# Supplementary material for: HIF1α-regulated glycolysis promotes activation-induced cell death and IFN-γ induction in hypoxic T cells
Source: Nat Commun. 2024 Oct 30;15:9394. doi: 10.1038/s41467-024-53593-8 (PMC11526104; doi:10.1038/s41467-024-53593-8)
Supplement: Supplementary file 7 — Reporting Summary [file 41467_2024_53593_MOESM7_ESM.pdf]

nature portfolio

Corresponding author(s): Lewis Z. Shi

Last updated by author(s): Jul 28, 2024

Reporting Summary

Nature Portfolio wishes to improve the reproducibility of the work that we publish. This form provides structure for consistency and transparency in reporting. For further information on Nature Portfolio policies, see our Editorial Policies and the Editorial Policy Checklist.

Statistics

For all statistical analyses, confirm that the following items are present in the figure legend, table legend, main text, or Methods section.

n/a	Confirmed	
☐	☒	The exact sample size (n) for each experimental group/condition, given as a discrete number and unit of measurement
☐	☒	A statement on whether measurements were taken from distinct samples or whether the same sample was measured repeatedly
☐	☒	The statistical test(s) used AND whether they are one- or two-sided<br>*Only common tests should be described solely by name; describe more complex techniques in the Methods section.*
☐	☒	A description of all covariates tested
☐	☒	A description of any assumptions or corrections, such as tests of normality and adjustment for multiple comparisons
☐	☒	A full description of the statistical parameters including central tendency (e.g. means) or other basic estimates (e.g. regression coefficient) AND variation (e.g. standard deviation) or associated estimates of uncertainty (e.g. confidence intervals)
☐	☒	For null hypothesis testing, the test statistic (e.g. F, t, r) with confidence intervals, effect sizes, degrees of freedom and P value noted<br>*Give P values as exact values whenever suitable.*
☒	☐	For Bayesian analysis, information on the choice of priors and Markov chain Monte Carlo settings
☒	☐	For hierarchical and complex designs, identification of the appropriate level for tests and full reporting of outcomes
☒	☐	Estimates of effect sizes (e.g. Cohen's d, Pearson's r), indicating how they were calculated

Our web collection on statistics for biologists contains articles on many of the points above.

Software and code

Policy information about availability of computer code

Data collection	1. All the flow cytometric data were acquired using the built-in software of the Attune NxT Flow Cytometer (Invitrogen, A24860) from Thermo Fisher.<br>2. All the animal studies were conducted in an IACUC-accredited animal facility at UAB, and tumor growth data were taken by regular measurements with a caliper.<br>3. All the Western blot results were obtained in the conventional film exposure methods in the dark room.<br>4. RNA-Seq was performed using the Illumina platform and then paired-end transcriptome sequences were mapped to the Mus musculus GRCm38 reference genome available on ENSEMBL using the STAR aligner (version 2.7.5a).<br>5. CUT&RUN reactions were conducted according to manufacturer's instructions (Epicypher 14-1048). Paired-end next-generation sequencing was conducted by Novogene Corporation, CA.<br>6. Extracellular flux OCR and ECAR were concurrently measured using the Wave software on a Seahorse XFe96 Analyzer (version 2.6.1).<br>7. 13C-labeled glucose metabolic flux was performed on an Agilent 7890A GC system equipped with a HP-5MS capillary column (30m, 0.25mm i.d., 0.25µm-phase thickness; Agilent J&W Scientific), connected to an Agilent 5977B Mass Spectrometer.
Data analysis	1. Flow cytometric data were analyzed using FlowJo version 10.8.1.<br>2. All the statistical analyses of the data were done using Prism-GraphPad version 9.4.0.<br>3. For RNA-seq data analysis, read counts per gene were calculated using htseq-count in the HTseq package (version 0.11.2). The analysis of differentially expressed genes (DEGs) between the WT and HIF1aKO T cells was performed using DESeq2 (version 1.34.0) in R (version 3.6.0). A volcano plot was used to show all upregulated and downregulated DEGs using the ggplot2 package (version 3.3.6). Heatmaps were made using the top 50 DEGs. Enriched Kyoto Encyclopedia of Genes and Genomes (KEGG) pathways of the DEGs were identified by enrichr package (version 3.0)<br>4. CUT&RUN sequenced reads were aligned to the mouse mm10 genome, and peaks of protein-DNA binding were visualized using the Integrative Genomics Viewer (IGV version 2.17.4).<br>5. Extracellular flux OCR and ECAR data were analyzed on Wave software (version 2.6.1) and the graphs were made by rism-GraphPad version 9.4.0.

6. Western blot quantification was performed on Fiji ImageJ version 1.52n.
7. Results from 13C-labeled glucose metabolic flux assay were analyzed using a compartmentalized metabolic network model using the Metran software, the high-resolution 13C metabolic flux model-13C-MFA.

For manuscripts utilizing custom algorithms or software that are central to the research but not yet described in published literature, software must be made available to editors and reviewers. We strongly encourage code deposition in a community repository (e.g. GitHub). See the Nature Portfolio guidelines for submitting code & software for further information.

Data

Policy information about availability of data

All manuscripts must include a data availability statement. This statement should provide the following information, where applicable:

- Accession codes, unique identifiers, or web links for publicly available datasets
- A description of any restrictions on data availability
- For clinical datasets or third party data, please ensure that the statement adheres to our policy

The RNA-seq data and Cut&Run data generated in this study have been deposited in the Gene Expression Omnibus (GEO) database under accession code GSE253090 [https://www.ncbi.nlm.nih.gov/geo/query/acc.cgi?acc=GSE253090]. Deposited data are publicly available. The remaining data in this study are available within the manuscript or Supplementary Data, with source data provided herein. Source data are provided with this paper.

Field-specific reporting

Please select the one below that is the best fit for your research. If you are not sure, read the appropriate sections before making your selection.

☒ Life sciences ☐ Behavioural & social sciences ☐ Ecological, evolutionary & environmental sciences

For a reference copy of the document with all sections, see nature.com/documents/nr-reporting-summary-flat.pdf

Life sciences study design

All studies must disclose on these points even when the disclosure is negative.

Sample size	We decide sample size based on our working experiences in this field, as reported in our previous publications (PMID: 36008408, PMID: 27498556, PMID: 27667683, and PMID: 31466995). For in vitro experiments, a minimum of 3 independent samples were used. For in vivo studies, at least 5 mice per group were anticipated in most of the experiments.
Data exclusions	All the animals were housed in animal facility for one week before tumor inoculation to exclude any unhealthy mice.
Replication	All the animal experiments were done at least twice, and each group had at least 5 mice to start with, although most of them were done for more than 3 times (as indicated). All the in vitro experiments were independently repeated, with similar results. All attempts at replication were successful.
Randomization	Animals were randomly allocated to groups with a table of random numbers.
Blinding	To avoid observer-expectation bias, tumor inoculation, tumor size measurement, and final data analysis were performed by different people.

Reporting for specific materials, systems and methods

We require information from authors about some types of materials, experimental systems and methods used in many studies. Here, indicate whether each material, system or method listed is relevant to your study. If you are not sure if a list item applies to your research, read the appropriate section before selecting a response.

Materials & experimental systems

n/a	Involved in the study	
☐	☒	Antibodies
☐	☒	Eukaryotic cell lines
☒	☐	Palaeontology and archaeology
☐	☒	Animals and other organisms
☒	☐	Human research participants
☒	☐	Clinical data
☒	☐	Dual use research of concern

Methods

n/a	Involved in the study	
☒	☐	ChIP-seq
☐	☒	Flow cytometry
☒	☐	MRI-based neuroimaging

Antibodies

Antibodies used	Flow antibodies used: Aqua fixation LIVE/DEAD™ Fixable Aqua Dead Cell Stain Kit (Thermo Fisher, #L34966; 1:200), anti-Mouse CD8 Brilliant Violet 785™ (clone 53-6.7, BD Biosciences #563332; 1:200), anti-Mouse CD4 Brilliant Violet 421™ (clone RM4-5, BioLegend #100544; 1:200), anti-Mouse CD45 PerCP-Cyanine5.5 (clone 30-F11, Thermo Fisher #45-0451-82; 1:200), Anti-Mouse CD45.1 PerCP-Cyanine5.5 (clone A20: BioLegend #110728; 1:200), Anti-Mouse CD45.2 Alexa Fluor® 700 (clone 104, BioLegend #109822; 1:200), Anti-Mouse TCRβ APC-Cy7 (clone H57-597, BioLegend #109220; 1:200), Anti-Mouse Perforin PE (clone S16009A, BioLegend #154306; 1:200), Anti-Mouse Granzyme B FITC (clone QA16A02, BioLegend #372206; 1:100), Anti-Mouse IFN-γ Brilliant Violet 650™ (clone XMG1.2, BioLegend #505832; 1:100), Anti-Mouse IL-2 Brilliant Violet 711™ (clone JES6-5H4, BioLegend #503837 ; 1:100), Anti-Mouse T-bet Brilliant Violet 711™ (clone 4B10, BioLegend #644820; 1:100), Anti-Mouse ICOS PerCP-Cyanine5.5 (clone 7E.17G9, BioLegend #117424; 1:200), Anti-Mouse CD25 APC (clone PC61.5, Thermo Fisher #17-0251-82; 1:200), Anti-Mouse FoxP3 eFluor™ 450 (clone FJK-16s, Thermo Fisher #48-5773-82; 1:100), Anti-Mouse HIF1α APC (clone Mgc3, Thermo Fisher #17-7528-82; 1:100), Anti-Mouse Ki-67 Alexa Fluor® 700 (clone SolA15, Thermo Fisher #56-5698-82; 1:100), Anti-Mouse RORγt Brilliant Violet 650™ (clone Q31-378 BD Biosciences #564722; 1:100), Anti-Human/Mouse Gata-3 eFluor™ 660 (clone TWAJ, Thermo Fisher #50-9966-42; 1:100), Anti-Human/Mouse Glut1 Alexa Fluor® 405 (clone EPR3915, Abcam #ab210438; 1:100), Anti-Human CD3 Super Bright™ 702 (clone OKT3, Thermo Fisher #67-0037-42; 1:200), Anti-Human IFN-γ Brilliant Violet 605™ (clone B27m, BD Biosciences #562974; 1:100), Anti-Human CD4 Brilliant Violet 650™ (clone L200, BD Biosciences #563737; 1:200).<br>The antibodies used for western blot are: HIF-1α (clone D2U3T, Santa Cruz Biotechnology 14179; 1:1000), Hif-2α (clone E2N9W, Cell Signaling Technology #57921; 1:1000), Glut1 (polyclonal antibody, Sigma #07-1401; 1:1000), Hk2 (clone C64G5, Cell Signaling Technology #2867; 1:1000), Ldha (Polyclonal antibody, Cell Signaling Technology 2012; 1:1000), Pkm2 (clone D78A4, Cell Signaling Technology 4053; 1:1000), Mct4 (clone D-1, Santa Cruz Biotechnology #sc-376140; 1:1000), Pdh (clone C54G1, Cell Signaling Technology #3205; 1:1000), phospho-Pdh (Ser293) (clone E4V9L, Cell Signaling Technology #37115; 1:1000), β-Actin (clone C-4, Santa Cruz Biotechnology #sc-47778 HRP; 1:10000).
Validation	We only order antibodies from highly-coveted reputable vendors (see above description) and all the antibodies that we used have been well-validated by the vendors, with validation statements available on manufacturer's websites, and commonly used in other studies.

Eukaryotic cell lines

Policy information about cell lines

Cell line source(s)	The B16-BL6 murine melanoma cells were kindly provided by Dr I. Fidler at MD Anderson Cancer Center. The chemically-induced murine bladder carcinoma MB49 cell line originated in a male C57BL/6 mouse was kindly provided by Dr A. Kamat at MD Anderson Cancer Center.
Authentication	All cell lines used are either from ATCC or commonly used by many investigators. They were originally obtained from Dr. Jim Allison and Dr. Pam Sharma labs when the senior author left MD Anderson Cancer Center to establish his own lab, with the permission from the original generators. All cell lines are not among the contamination list of the International Cell Line Authentication Committee.
Mycoplasma contamination	All cells are regularly tested and remain free of mycoplasma and other pathogens that are regulated by the IACUC committee at UAB.
Commonly misidentified lines (See ICLAC register)	We did not use any misidentified lines.

Animals and other organisms

Policy information about studies involving animals; ARRIVE guidelines recommended for reporting animal research

Laboratory animals	Seven-week-old female and male C57BL/6 (Stock No: 000664) were purchased from The Jackson Laboratory (Bar Harbor, ME). Hif1α-/- and Vhl-/- mice were bred in our animal facility, with the original breeders procured from The Jackson Laboratory (Stock #: 007561, Stock #: 002014, and Stock #: 022071). All mice were housed in specific pathogen-free conditions in the animal facility of The University of Alabama at Birmingham (UAB) under 12 hours/12 hours light/dark cycle, ambient room temperature (22 °C) with 40%-70% humidity.
Wild animals	We did not use wild animals in this study.
Field-collected samples	We did not have field-collected samples; all data were generated in my laboratory.
Ethics oversight	All animal protocols were approved by Institutional Animal Care and Use Committee (IACUC) at UAB (APN-21945).

Note that full information on the approval of the study protocol must also be provided in the manuscript.

Flow Cytometry

Plots

Confirm that:

☒ The axis labels state the marker and fluorochrome used (e.g. CD4-FITC).

☒ The axis scales are clearly visible. Include numbers along axes only for bottom left plot of group (a 'group' is an analysis of identical markers).

☒ All plots are contour plots with outliers or pseudocolor plots.

☒ A numerical value for number of cells or percentage (with statistics) is provided.

Methodology

Sample preparation	Tumors were collected in ice-cold RPMI 1640 containing 2% FBS and minced into fine pieces, followed by digestion with 400 U/mL collagenase D (Worthington Biochemical Corporation, LS004186) and 20 µg/mL DNase I (Sigma, 10104159001) at 37 C for 40 min with periodic shaking. EDTA (Sigma, 1233508) was then added to the final concentration of 10 mM to stop digestion. Cell suspensions were filtered through 70 µM cell strainers, and TILs were obtained by collecting the cells in the interphase after Ficoll (MP Biomedicals, 091692254). Spleens and DLNs were collected in ice-cold HBSS containing 2% FBS to prepare single cell suspensions, after lysis of red blood cells and filtering with nylon mesh. TILs, splenocytes, and DLN cells were resuspended in complete Click's culture medium for flow cytometric analyses. Activted T cells were harvested from the culture plates and gently washed off. Cells were either directly used in apoptotic analyses or ficolled to get rid of dead cells, followed by surface staining with the said antibodies in DPBS containing 2% BSA for 30 min on ice. To analyze FoxP3, T-bet, RORrt, and HIF1a, following surface staining, cells were fixed using the Foxp3/Transcription Factor Staining Buffer Set (Invitrogen, 00-5523-00) and stained for those markers, according to the manufacturer's instructions. To detect intracellular cytokines, cells were briefly stimulated for 4-5 h with PMA (final concentration: 50 ng/mL) plus ionomycin (final concentration: 1 uM) in the presence of monensin (for the last 2 h). Stimulated cells were stained with surface markers, fixed using the BD Cytofix/Cytoperm Plus Fixation/Permeabilization Kit (BD Biosciences, 554715), and stained for cytokines, according to the manufacturer's instructions.
Instrument	Attune NxT Flow Cytometer (Invitrogen, A24860)
Software	Data were acquired using the built-in acquisition software of Attune Flow Cytometer and analyzed using Flowjo software version 10.8.1 (Tree Star).
Cell population abundance	The abundance of the relevant cell populations were presented in percentages, which were clearly defined using well-recognized markers.
Gating strategy	The preliminary FSC/SSC gates representing either total cells or lymphocytes were further gated on singlets (FSC-H vs FSC-A) and then live cells based on the staining with a fixable live/dead dye. The positive vs negative gating was based on no-stain controls (FMO) and the senior author's many years of working experiences in the field.

☒ Tick this box to confirm that a figure exemplifying the gating strategy is provided in the Supplementary Information.
